# Multimodal brain imaging effect of “Adjust Zang-fu and Arouse Spirit” electroacupuncture on diabetic cognitive impairment: study protocol of a randomized, sham-controlled pilot trial

**DOI:** 10.1186/s13063-021-05842-0

**Published:** 2021-11-25

**Authors:** Lin Yao, Mengyuan Li, Shunan Sun, Ming Xu, Shuo Yu, Ziyang Zhang, Liying Zhang, Haizhu Zheng, Zhen Zhong, Shiqi Ma, Haipeng Huang, Hongfeng Wang

**Affiliations:** Changchun University of Chinese Medicine, No.1035 Boshuo Road, Nanguan District, Changchun, Jilin, 130117 China

**Keywords:** Electropuncture, Diabetic Cognitive Impairment, Multimodal brain imaging; Randomized controlled trial, Study protocol

## Abstract

**Background:**

Diabetic cognitive impairment (DCI) is a serious chronic complication caused by diabetes. The pathogenesis of DCI is complex, but brain nerve injury and brain nerve cell apoptosis are important pathological changes. Multimodal brain imaging is one of the most important techniques to study the neural mechanism of the brain. For the clinical treatment of DCI, there is no effective targeted Western medicine and a lack of clear drug intervention methods. Therefore, there is an urgent need to find effective complementary and alternative methods and clarify their mechanism. This research seeks to explore the multimodal brain imaging effect of “Adjust Zang-fu and Arouse Spirit” electroacupuncture for DCI.

**Methods:**

This clinical research will be a randomized, sham-controlled pilot trial. Eligible participants will be randomly assigned to the intervention group (*n* = 60) and the control group (*n* = 30). The intervention group will be divided into the “Adjust Zang-fu and Arouse Spirit” electroacupuncture group (*n* = 30) and sham electroacupuncture group (*n* = 30). All participants will continue to receive routine hypoglycemic therapy. The treatment period is the same in both groups. The primary outcomes include functional magnetic resonance imaging (fMRI), magnetic resonance spectroscopy (MRS), Montreal Cognitive Assessment Scale (MoCA), and Clinical Dementia Rating (CDR). The secondary outcomes include blood glucose and blood lipid tests, Instrumental Activities of Daily Living Scale (IADL), Hachinski Ischemic Scale (HIS), Self-Rating Anxiety Scale (SAS), and Self-Rating Depression Scale (SDS). Outcomes will be assessed at baseline and before and after treatment, and adverse events will be examined. Inter- and intragroup analyses will be performed.

**Discussion:**

This randomized controlled study, combined with multimodal brain imaging techniques and a clinical evaluation scale, was designed to explore the mechanism of “Adjust Zang-fu and Arouse Spirit” electroacupuncture in improving the central nervous system in DCI.

**Trial registration:**

Chinese Clinical Trial Registration ChiCTR2000040268. Registered on 26 November 2020

## Background

Diabetes mellitus (DM) is a chronic disease that causes huge economic and social burdens. In recent years, a large number of studies have proven that DM has become an independent risk factor for cognitive impairment. The incidence of cognitive impairment is 1.5 times higher in type 2 diabetes mellitus (T2DM) patients than in non-DM patients [1], with 60 to 70% of T2DM patients having varying degrees of cognitive impairment [2]. Diabetic cognitive impairment (DCI) is a serious chronic complication caused by diabetes and is mainly manifested as deficiency of attention, visual structure, learning function, verbal memory, and calculation ability [3]. It is a state of cognitive impairment between brain aging and dementia that belongs to the early stage of Alzheimer's disease (AD) [4]. Serious cognitive deficits, especially in executive function, impact the self-care and medical compliance of these patients. DCI brings a serious burden to patients’ families and society, and thus effective prevention, early diagnosis, management, and treatment of DCI have become the focus of clinical research.

Studies have shown that patients with DCI have brain atrophy, neurofibril tangles, senile plaques, regional neuronal apoptosis, and other pathological changes [5, 6]. Although the pathogenesis of DCI is complex, brain nerve injury and brain nerve cell apoptosis are important pathological changes of DCI that result in brain functional area atrophy, functional connection network changes, and other pathological changes [7]. In recent years, the rapid development of multimodal brain imaging technology has provided new research ideas and techniques for scholars to carry out DCI research at the living human level. Several studies using functional magnetic resonance imaging (fMRI) have found that the brain function and structure of patients with DM show changes similar to those of dementia. For example, the volume and thickness of the hippocampus and other brain regions, such as the hippocampus, entorhinal cortex and medial temporal lobe, are atrophied and are closely related to emotion, speech, and memory [8–10]; the connectivity of the default mode network and control network is impaired [11]; the expression levels of N-acetylaspartate and γ-aminobutyric acid in the brain are decreased; and myo-inositol, glutamate and glycine are increased [12]. Therefore, the close combination of brain imaging with pathophysiology and clinical research will be an important means to uncover the mystery of DCI, AD and other diseases.

At present, the exact pathogenesis and effective treatment methods of DCI are in the exploratory stage. There is no specific Western medicine for the effective treatment of DCI, and there is a lack of clear drug intervention methods. Acupuncture is an important method for the prevention and treatment of DM and its complications, which can control blood glucose and regulate brain activation areas and abnormal brain functional connections. Studies have confirmed that acupuncture combined with metformin therapy can more effectively improve the level of fasting blood glucose in patients with T2DM [13]. Many studies have found that acupuncture can alter the changes in brain regions and functional connectivity related to cognition in patients with AD and MCI [14–16]. Therefore, in this proposed study, we aimed to (1) carry out a randomized, pseudocontrolled clinical trial comprehensively using multimodal brain imaging techniques to reveal the changes in brain structure and function in patients with DCI, (2) clarify the brain function network effect of “Adjust Zang-fu and Arouse Spirit” electroacupuncture to prevent and improve cognitive dysfunction in patients with DCI, and (3) analyze the correlation between the changes in clinical cognitive function and the changes in brain structure and function.

## Methods/design

### Study design

This is a randomized controlled trial. The protocol was approved by the Medical Ethics Committee of the Third Affiliated Hospital of Changchun University of Chinese Medicine (approval No. of ethics committee: CZDSFYLL2020-001-01) and registered at the Chinese Clinical Trial Registry (registration number: ChiCTR2000040268, protocol version number: V3.0, 02 October 2020.).

In this study, the tested patients will be randomly divided into two groups: an intervention group and a control group. Patients who meet the inclusion and exclusion criteria will first complete informed consent and then be randomized and stratified according to the clinical treatment. The intervention group will be divided into the “Adjust Zang-fu and Arouse Spirit” electroacupuncture group, which will be treated with electroacupuncture on a fixed prescription of acupoints, and the sham electroacupuncture group, which will be treated with electroacupuncture on nonacupuncture points and a sham instrument.

### Setting of the study

**Fig. 1:**
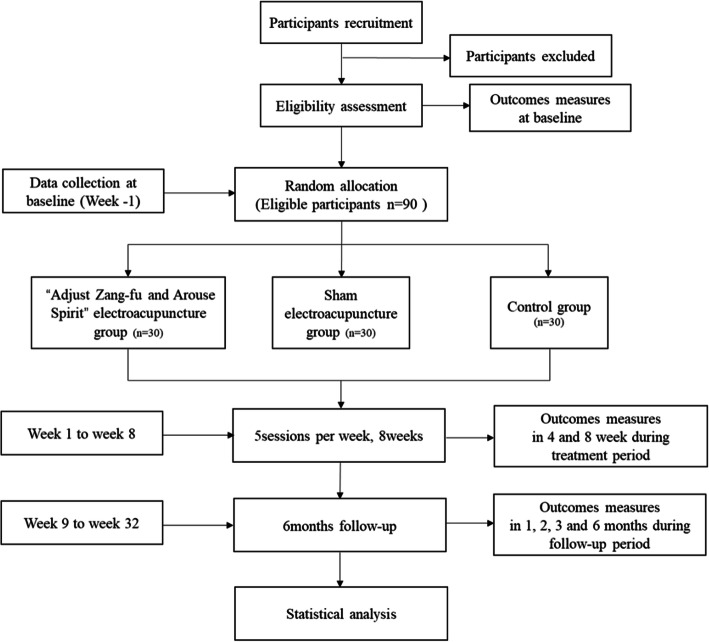
Flow diagram of the study design

This clinical trial will be conducted at the third Affiliated Hospital of Changchun University of Chinese Medicine (Changchun, China). Eligible participants will be randomly assigned to the intervention group (*n* = 60) and the control group (*n* = 30). The intervention group will be divided into the “Adjust Zang-fu and Arouse Spirit” electroacupuncture group (*n* = 30) and sham electroacupuncture group (*n* = 30). The total observation period will include a − 3~0-day baseline selection period, an 8-week treatment period, and a 6-month follow-up period. They will receive 40 sessions of electroacupuncture or sham electroacupuncture treatment over 8 weeks (five sessions per week). Assessments will be conducted at baseline and at 4 and 8 weeks after randomization, as well as at 1, 2, 3, and 6 months after treatment. Figure 1 illustrates the study flowchart. This protocol will be reported according to the SPIRIT 2013 [17] statement of recommendations for intervention trials: Defining Standard Protocol Items for Clinical Trials. The trial process was designed according to the STRICTA 2015 [18] report (Table 1).

**Table 1:** Acupuncture treatment details based on the STRICTA 2015 checklist

Item	Item number	Detail
**1.Acupuncture rationale**	1a) Style of acupuncture	Traditional Chinese Medicine
	1b) Reasoning for treatment	The treatment protocol was based on traditional acupuncture theory, previous studies guidelines, and the consensus of acupuncturists from the third Affiliated Hospital of Changchun University of Chinese Medicine
	1c) Extent to which treatment was varied	All participants will receive standardized treatment.
**2. Details of needling**	2a) Number of needle insertions per subject per session	16
	2b) Names of points used	Nine fixed points: GV20, GV24, BL13, BL20, BL23, ST36, SP6, LI4, LR3
	2c) Depth of insertion, based on a specified unit of measurement	From 0.5 to 1.5 cun
	2d) Response sought (e.g., *de qi* or muscle twitch response)	*De qi* sensation
	2e) Needle stimulation (e.g., manual, electrical)	Electrical stimulation: the electric stimulators (SDZ-V electroacupuncture apparatus, Suzhou Medical Appliance Factory, China) will be attached to needles at the GV20-GV24, unilateral BL13-BL23 and SP6-LR3 points, and a continuous wave of 2 Hz/100 Hz frequency, and an intensity of 0.1–1.0 mA will be applied
	2f) Needle retention time	30 min
	2g) Needle type	Sterile disposable stainless steel needles of various lengths and diameters (Hwato Brand, Suzhou Medical Appliance Factory, China; 0.3 mm × 25 mm/0.3 mm × 40 mm)
**3. Treatment regimen**	3a) Number of treatment sessions	40 treatment sessions in both acupuncture and sham acupuncture groups
	3b) Frequency and duration of treatment sessions	Five times a week, followed by a 2-day rest interval, for 8 successive weeks
**4. Other components of treatment**	4a) Details of other interventions administered to the acupuncture group (e.g., moxibustion, cupping, herbs, exercises, lifestyle advice)	No other interventions during the study period were allowed. Light diet and do not eat beef, lamb, seafood, mushrooms, and so on
	4b) Setting and context of treatment, including instructions to practitioners and information and explanations to patients	The study will be conducted at the acupuncture and massage center of the third Affiliated Hospital of Changchun University of Chinese Medicine. All patients will be admitted to the acupuncture therapeutic room for treatment. All information except patient allocated group will be provided to participants
**5.Practitioner background**	5) Description of participating acupuncturists	Acupuncturists who are registered Chinese Medicine Practitioners in China and have at least 3 years’ clinical experience in acupuncture practice. Besides, they went through training classes before this trial and simulation to ensure that they are able to provide identical acupuncture treatment in accordance with a pre-defined protocol
**6. Control interventions**	6a) Rationale for the control or comparator in the context of the research question, with sources that justify this choice	Based on traditional acupuncture theory, previous studies, and the consensus of acupuncturists from the third Affiliated Hospital of Changchun University of Chinese Medicine
	6b) Precise description of the control or comparator. If sham acupuncture or any other type of acupuncture-like control is used, provide details as for Items 1 to 3 above	For the Sham-electroacupuncture group: participants will receive superficial non-acupoint sham- electroacupuncture 40 sessions over 8 weeks. Non-acupoints will be needled through adhesive pads to the skin (for the fixation of needles) without penetrating the skin using 1 cun blunt needles. The needle parameters and treatment regimen are the same as for the electroacupuncture group. When turned on, electric acupuncture apparatus looks normal but has no current output

*Abbreviation*: STRICTA, Standards for Reporting Interventions in Clinical Trials of Acupuncture

### Participants

We will recruit 90 participants from the third Affiliated Hospital of Changchun University of Chinese Medicine (Changchun, China). Before treatment, we will perform glycosylated hemoglobin A1 (HbA1c) tests, blood lipid profile indices (total cholesterol (TC), triglycerides (TGs), high-density lipoprotein cholesterol (HDL-C), and low-density lipoprotein cholesterol (LDL-C)), fMRI screens and scale assessments for the participants to select the patients to be included. For outpatients who meet the inclusion and exclusion criteria and wish to participate in the study, we will fully communicate with patients and their family members face-to-face about the trial to ensure that the patients are able to participate throughout the study.

### Recruitment

To recruit participants, we will publicize the study through recruitment posters, scrolling electronic screens, leaflets, and WeChat public platforms. During the recruitment process, we will fully communicate with patients, clearly informing them of the purpose, significance, trial process, treatment method, and possible benefits and risks of this study. Then, we will inform patients and their guardians of the contents of the informed consent form one-by-one. When patients and their guardians completely agree with all the items in the consent form, they will sign it of their own free will.

### Inclusion criteria

Eligible participants for DCI subjects should meet the following inclusion criteria: (1) the 2020 American Diabetes Association Standards of Medical Practice for diabetes with a duration of ≥ 2 years; (2) signs or symptoms of memory loss; (3) Montreal Cognitive Assessment Scale (MoCA) score < 26 points, Clinical Dementia Rating (CDR) ≥ 0.5 points; (4) activities of daily living (ADLs) not significantly affected, and instrumental ability of daily living (IADL) score ≥ 16 points; (5) aged 50–70 years, with more than 6 years of education, able to complete a cognitive assessment; (6) sufficient visual and auditory discrimination to undergo neuropsychological testing; (7) no evidence of infection, infarction, or other focal injury, or associated clinical symptoms, as detected by transcranial CT or MRI in the 12 months prior to screening; allows for white matter lesions or cavitary infarcts in noncritical brain regions without affecting the subject’s cognitive function; and (8) signed informed consent, volunteered to participate in the study, and were able to cooperate with the physician in completing the clinical study. Eligible participants who meet the following inclusion criteria as healthy subjects: (1) conditions such as age and education that are comparable to other groups of subjects; (2) absence of significant neurological foci and absence of associated disorders that could cause cognitive impairment, as determined by cranial MRI; (3) no religious beliefs, no bad habits, such as tobacco, alcohol, coffee, and tea; (4) no job or life stress during the observation period; (5) in good health, free of cardiovascular and other organic diseases; (6) total MoCA score ≥ 26 points; and (7) signed informed consent, volunteered to participate in the study, and were able to cooperate with the physician in completing the clinical study.

### Exclusion criteria

Participants meeting any of the following criteria will be excluded: (1) sensitivity to acupuncture; (2) subjects with significant auditory, visual, or speech impairments affecting cognitive function testing; (3) acute complications of diabetes such as diabetic ketoacidosis and hypertonic coma within the last 3 months, and significant cardiopulmonary, hepatic, renal, and hematologic diseases that may affect cognitive function; (4) history of previous cerebrovascular disease such as cerebral infarction, cerebral hemorrhage, or other focal injury that affects cognition; vascular dementia (Hachinski Ischemia Index score ≥ 7 points) and neurological disorders that can cause dementia such as Alzheimer’s disease and Parkinson’s disease; history of severe traumatic brain injury with persistent neurological deficits or known structural brain abnormalities; (5) depression within the past 2 years with an Anxiety Self-Assessment Scale (SAS) or Depression Self-Assessment Scale (SDS) score > 50 points; (6) history of alcohol, tobacco, drug abuse, or dependence within the past 2 years; (7) use of medications in the following categories within 30 days prior to screening for cognitive impairment: medications for Alzheimer's disease or dementia, antiparkinsonian medications, short-acting anxiolytics, neuroleptic or analgesic medications, antiepileptic medications, hormones, or medications with significant cholinergic or anticholinergic side effects; (8) more pronounced cerebral white matter lesions or cavitary infarction (subject’s age-related white matter change (ARWMC) score ≥ 2 points); and (9) contraindications to MRI (claustrophobia, pacemaker implants or surgical metal plate in the body).

### Withdrawal criteria

Participants meeting any of the following criteria will be withdrawn by the unanimous decision of the two researchers: (1) participant’s decision to drop out of the study at any time for any reason; (2) death or other major adverse events or accidents occur due to disease development; (3) participants display poor compliance with the clinical trial protocols, an unwillingness to continue in the study, or requests to withdraw from the study; and (4) participants are noncompliant with the prescribed treatment, are not treated as prescribed, or have incomplete observation data affecting the evaluation.

### Randomization, concealment of allocation, and blinding

Participants who meet the inclusion criteria will voluntarily sign an informed consent form and will be eligible for random assignment. They will be assigned randomly to the “Adjust Zang-fu and Arouse Spirit” electroacupuncture group or sham electroacupuncture group. The randomization sequence will be generated by a statistician not involved in the intervention or outcome evaluation using IBM® SPSS® Statistics version 24.0 (IBM Research Corporation, USA). The randomization numbers and group assignments will be sent immediately to the independent assessor by another research coordinator via an encrypted electronic file. As much as possible, evaluators will be prevented from communicating with the patients to avoid bias. This process will ensure that randomization concealment will be competent and unaffected by the participants or practitioners.

Due to the particularity of electroacupuncture therapy, double-blinding of the therapist and patient is not feasible. Before signing the informed consent form, patients will be told that they will receive electroacupuncture treatment. Participants will be unaware of their treatment assignment. The therapist will not be blinded but will not assess the effectiveness of the treatment. Outcome assessors, data managers and statisticians will be blinded to treatment allocation. Prior to the start of the trial, all researchers will be trained to ensure the successful implementation of the blinding procedure.

### Interventions

#### Blood glucose control

The subjects in the “Adjust Zang-fu and Arouse Spirit” electroacupuncture group and the sham acupuncture group will receive routine hypoglycemic therapy of Western medicine, and their blood glucose will be strictly monitored to keep their blood glucose stable.

#### Electroacupuncture intervention

In this trial, treatment strategies were developed by consensus with experienced acupuncture practitioners and radiologists. There are two groups: “Adjust Zang-fu and Arouse Spirit” electroacupuncture group or sham electroacupuncture group. The location and manipulation of fixed acupoints and nonacupoints are shown in Table 2. All electroacupuncture treatment sessions will be performed by four Chinese Medicine Practitioners acupuncturists registered in China with at least 3 years of clinical experience in acupuncture practice. Acupuncturists will also receive training prior to this trial. The training course will include the location of acupoints, needle manipulation skills, and communication skills. When the acupuncturists are able to pass the trial training examination, they will be admitted to participate in the trial.

**Table 2:** Locations and manipulations of the acupuncture points selected in this study

Group	Acupoints	Location	Manipulation
“Adjust Zang-fu and Arouse Spirit” electroacupuncture group	Baihui (GV20)	In the head, the front of the forehead is straight up to 5 cun	Subcutaneous insertion to a depth of 0.5-0.8 cun (12.5–20 mm) with manipulation for the de-qi
	Shenting (GV24)	In the head, the front of the forehead is straight up to 0.5 cun	Subcutaneous insertion to a depth of 0.5–0.8 cun (12.5–20 mm) with manipulation for the de-qi
	Feishu (BL13)	In the spinal region, under the spinous process of the third thoracic vertebrae, 1.5 cun beside the posterior midline	Oblique insertion to a depth of 0.5–0.8 cun (12.5–20 mm) with manipulation for the de-qi
	Pishu (BL20)	In the spinal region, under the spinous process of the 11th thoracic vertebrae, 1.5 cun beside the posterior midline	Oblique insertion to a depth of 0.5–0.8 cun (12.5–20 mm) with manipulation for the de-qi
	Shenshu (BL23)	In the spinal region, under the spinous process of the second lumbar spine, 1.5 cun beside the posterior midline	Puncture perpendicularly to a depth of 0.5–1.0 cun with manipulation for the de-qi.
	Zusanli (ST36)	Three inches below the patella between the anterior tibia and the extensor digit rum longus muscle	Puncture perpendicularly to a depth of 1–1.5 cun with manipulation for the de-qi
	Sanyinjiao (SP6)	On the medial side of the leg, 3 cun above the tip of the medial malleolus, posterior to the medial border of the tibia	Puncture perpendicularly to a depth of 1.0 cun with manipulation for the de-qi
	Hegu (LI4)	On the back of the hand, at the midpoint of the radial side of the second metacarpal bone	Puncture perpendicularly to a depth of 0.5–1.0 cun with manipulation for the de-qi
	Taichong (LR3)	In the posterior recess of the 1st metatarsal space	Puncture perpendicularly to a depth of 0.5–0.8 cun (12.5–20 mm) cun with manipulation for the de-qi
Sham-electroacupuncture group	Beside GV20	It is 0.5 cun (12.5 mm) to the right of GV20	Needled through adhesive pads to the skin (for the fixation of needles) without penetrating the skin and manipulation for the de-qi
	Beside GV24	It is 0.5 cun (12.5 mm) to the right of GV24	Needled through adhesive pads to the skin (for the fixation of needles) without penetrating the skin and manipulation for the de-qi
	Beside BL13	It is located 1 cun outside BL13	Needled through adhesive pads to the skin (for the fixation of needles) without penetrating the skin and manipulation for the de-qi
	Beside BL20	It is located 1 cun outside BL20	Needled through adhesive pads to the skin (for the fixation of needles) without penetrating the skin and manipulation for the de-qi
	Beside BL23	It is located 1 cun outside BL23	Needled through adhesive pads to the skin (for the fixation of needles) without penetrating the skin and manipulation for the de-qi
	Beside ST36	It is located 1 cun backward of ST36	Needled through adhesive pads to the skin (for the fixation of needles) without penetrating the skin and manipulation for the de-qi
	Beside SP6	It is located 1 cun backward of P6	Needled through adhesive pads to the skin (for the fixation of needles) without penetrating the skin and manipulation for the de-qi
	Beside LI4	It is located 1 cun in front of LI4	Needled through adhesive pads to the skin (for the fixation of needles) without penetrating the skin and manipulation for the de-qi
	Beside LR3	It is located 1 cun backward of LR3	Needled through adhesive pads to the skin (for the fixation of needles) without penetrating the skin and manipulation for the de-qi

#### “Adjust Zang-fu and Arouse Spirit” electroacupuncture treatment group

When the patients are in a comfortable sitting position, all acupoints will be punctured by filiform needles (0.3 mm × 25 mm/0.3 mm × 40 mm, Hwato Brand, Suzhou Medical Appliance Factory, China). Then, lifting, thrusting, twisting, and rotating manipulations will be applied on the needles until the *de-qi* sensation is achieved. After achieving the *de-qi* sensation, electric stimulators (SDZ-V electroacupuncture apparatus, Suzhou Medical Appliance Factory, China) will be attached to needles at the GV20-GV24, unilateral BL13-BL23 and SP6-LR3 points, and a continuous wave of 2 Hz/100 Hz frequency and an intensity of 0.1–1.0 mA will be applied.

#### Sham-electroacupuncture treatment group

Nonacupoints will be needled through adhesive pads to the skin (for the fixation of needles) without penetrating the skin using 1 cun blunt needles. The sham electrode lines look identical to the real lines but with the inner metal wire cut off. The electric stimulators will connect the beside GV20-beside GV24, unilateral beside BL13-beside BL23, and beside SP6-beside LR3. The needle parameters were the same as those for the electroacupuncture group. When turned on the electroacupuncture instrument, the electric acupuncture apparatus looks normal but has no current output.

For both groups, the needles will be retained for 30 min for each treatment session. The participants will be treated with electroacupuncture 5 times a week, followed by a 2-day rest interval for 8 successive weeks, with 40 sessions for each patient in total.

### MRI scanning procedure

The Siemens 3.0 T Magnetom Verio MRI system (Siemens Medical, Erlangen, Germany) with a 32-channel head coil will be used to obtain MRI data at the Affiliated Hospital of Changchun University of Chinese Medicine (Changchun, China). To avoid head movement, foam pads will be used to fix the participants’ heads. The fMRI data will be acquired with a single-shot gradient recalled echo planar imaging sequence with the following parameters: repetition time (TR)/echo time (TE) =3000 ms/30 ms, flip angle = 85, field of view (FOV) = 220 mm × 220 mm and slice thickness = 3 mm. Magnetic resonance spectroscopy (MRS) data will be acquired with 3D multivoxel proton magnetic resonance spectroscopy with the following parameters: repetition time (TR)/echo time (TE) =1750 ms/144 ms, slice thickness = 5 mm and repeat time = 128.

### Outcome measures

**Fig. 2:**
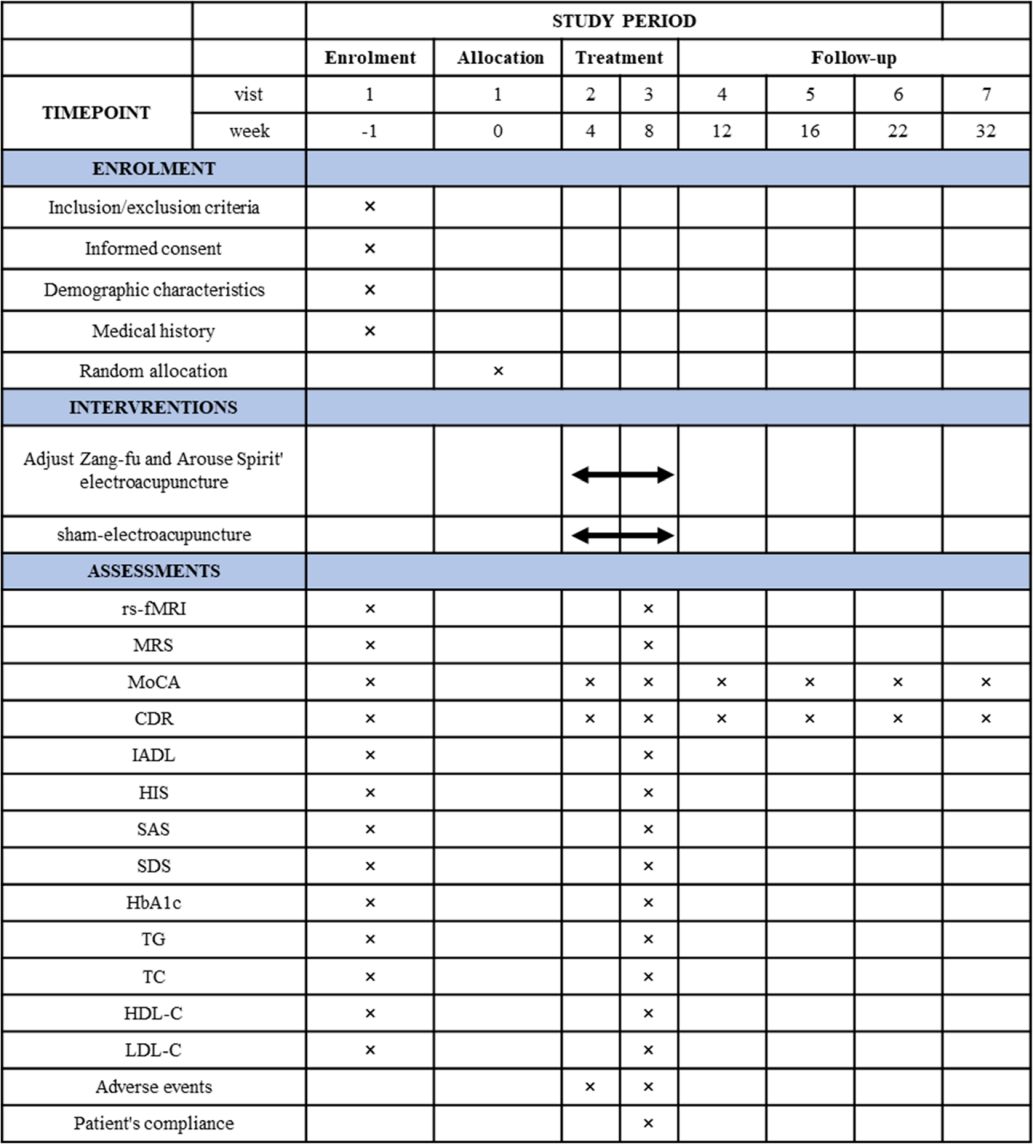
Timetable of treatment and outcome collection

The time schedule of enrollment, interventions, and assessments of participants are shown in Fig. 2. The following outcomes will be assessed by independent assessors.

#### Primary outcomes

The primary outcomes include whole-brain structure and function evaluated using fMRI, metabolites in the basal ganglia evaluated using MRS, and cognitive function assessed using the MoCA and CDR.

##### Montreal Cognitive Assessment Scale (MoCA)

This scale is an assessment tool for rapid screening of cognitive abnormalities. It is a validated scale for MCI in diabetes in China [19]. It includes 8 dimensions (attention and concentration, executive function, memory, language, visual structure skills, abstract thinking, calculation and orientation), with a total score of 30 and ≥ 26 points being normal. It is suitable for clinical applications because of its high sensitivity, coverage of important cognitive domains, and short testing time. However, it is also affected by education level, differences in cultural background, the examiner’s skills and experience in using MoCA, the environment of the examination, and emotional and mental state of the test taker will all affect the score. To correct the bias caused by education level, the total score is increased by 1 point for subjects with less than 12 years of education. This can better correct the bias caused by low education levels.

##### Clinical Dementia Rating (CDR)

This scale is a global dementia grading scale that assesses cognitive changes and determines the presence of dementia [20]. The scope of the assessment includes three cognitive domains (memory, orientation, judgment and problem solving skills) and three related activities of daily living skills (social interaction, family life, hobbies and self-care). The severity of dementia is quantified from very mild (CDR 0.5) to mild (CDR 1), moderate (CDR 2), and severe (CDR 3).

#### Secondary outcomes

The secondary outcomes include changes in blood glucose and blood lipids, assessed using HbA1c, TC, TGs, HDL-C, and LDL-C; activities of daily living, assessed using IADL; exclusion of patients with vascular dementia, assessed using Hachinski Ischemic Scale (HIS); and exclusion of patients with abnormal psychiatric behavior assessed using SAS and SDS.

##### Instrumental Activities of Daily Living Scale (IADL)

This scale may identify incipient decline-physical, cognitive, or both-in an older adult who might otherwise appear capable and healthy. It assesses a person’s ability to perform tasks including telephoning, shopping, food preparation, housekeeping, laundering, use of transportation, use of medicine, and financial behavior [21].

##### Hachinski Ischemic Scale (HIS)

This scale is composed of 13 items, which are derived from clinical practice experience. It needs to be judged by combining the patient’s history, clinical symptoms, signs, and auxiliary examination results. It can be simple, easy, and effective to distinguish vascular dementia from Alzheimer’s disease. HIS ≥ 7 points supports vascular dementia, and ≤ 4 points supports Alzheimer’s disease [22].

##### Self-Rating Anxiety Scale (SAS)

The scale includes 20 entries divided into four grade ratings according to the frequency of anxiety status in the past 7 days. The standard cutoff scores used define < 50 as no anxiety, 50–59 as minimal to mild anxiety, 60–69 as moderate to marked anxiety, and ≥ 70 as severe anxiety [23].

##### Self-rating depression scale (SDS)

The scale includes 20 entries divided into four grade ratings according to the frequency of depression status in the past 7 days. The standard cutoff scores used define < 50 as no depression, 50–59 as minimal to mild depression, 60–69 as moderate to marked depression, and ≥ 70 as severe depression [24].

### Adverse events

The participants will be requested to voluntarily report information about adverse events, including possible discomfort symptoms during acupuncture treatment, such as fainting, bleeding, local hematoma, and unbearable prickling, and continuous severe pain after acupuncture. The acupuncturists will be requested to report adverse events related to acupuncture, such as sticking of the needle, broken needles, and bent needles. All adverse events will be fully recorded on the adverse event pages of the case report forms (CRFs). If any serious adverse reactions occur, emergency medical assistance will be sought, and all details will be noted. The outcome measurement will be assessed at 1 and 2 weeks after randomization.

### Sample size estimation

The sample size was calculated based on the MoCA scale score as the primary outcome. The sample size was calculated based on the noninferiority comparison of the two groups: *α* = 0.025, 1−*β* = 0.80, *Δ* = − 0.1, which was calculated by PASS software with *n* ≈ 24. Because there was partial shedding in the treatment process, the shedding rate was 20%, and thus, it was appropriate to increase the sample size and take *n* = 30 cases per group. Therefore, 30 cases per group were needed, totaling 90 patients in 3 groups.

### Data collecting and monitoring

The Data Monitoring Committee is composed of members of the Medical Ethics Committee of the Third Affiliated Hospital of Changchun University of Traditional Chinese Medicine, and the committee has determined that there is no conflict of interest with this experiment. The data will be recorded on the paper CRFs by the designated result evaluator and double-entered into the electronic CRFs, which are established and monitored by the Medical Ethics Committee of the Third Affiliated Hospital of Changchun University of Traditional Chinese Medicine. A data acquisition and management system was established, including the dual input of data entry personnel and researchers, and checked by a third person to ensure the objectivity, authenticity, and accuracy of the research data. Monitors will audit the data every 3 months. This process will be independent from investigators. Acupuncturists and statisticians will not be able to access the data during the evaluation process. The researcher with the approved proposal will be permitted to access the final dataset by contacting the corresponding authors.

### Statistical analysis

#### Clinical data analysis

Statistical analysis of the clinical outcomes will be performed using SPSS 24.0 statistical software. All statistical tests will be bilateral, and *p* < 0.05 will be considered statistically significant. For clinical information, data will be presented as the mean ± standard deviation. Student’s *t* test and the chi-square test will be used to compare group differences at baseline. A paired *t* test will be used to compare the clinical outcomes within the groups. Analysis of variance and the Wilcoxon rank-sum (if normality is violated) will be used to compare the clinical feature changes between groups. The clinical data, including MoCA, CDR, HbA1c, TC, TGs, HDL-C, and LDL-C, will be converted to domain z scores for correlational analysis with imaging data. Missing data for the primary outcome will be imputed using the multiple imputation method.

#### Neuroimaging data analysis

Neuroimaging data processing and statistical analysis were carried out by the individualized Neuroimaging Research Laboratory of Martinos Neuroimaging Center, Massachusetts General Hospital, Harvard Medical School, USA.

The structural MRI data will be analyzed using the VBM toolbox within SPM12. The steps include interlayer time difference correction, spatial difference correction, head motion correction, standardization, and Gaussian smoothing. In the head motion correction process, the images of the subjects whose translation is less than 1 mm and rotation movement is less than 2° will be involved in the follow-up analysis. The fMRI data will be analyzed using the CIVET-pipeline toolbox and SurfStat in MATLAB 2018b. The steps include removing high-frequency components by a bandpass filter, removing the average signal of the whole brain and the signals of the ventricle and white matter, and then registering the data to the surface space of FreeSurfer. T tests and repeated-measures analysis of variance will be conducted to investigate the differences in brain regions in each group. Pearson correlation analysis will be used to test the correlation between fMRI data and clinical variables. According to the results of MRS scanning, the concentration of target metabolites will be statistically analyzed. A paired *t* test will be used to compare within the groups. Analysis of variance and the Wilcoxon rank-sum (if normality is violated) will be used to compare between groups.

### Quality control

Acupuncture, neurology, and methodology experts reviewed and revised the experimental protocol many times. Before the trial, all staff will attend a series of training sessions. These sessions will ensure that relevant personnel are fully aware of the study protocol and the standard operating procedures for the study. During the trial, supervisors will review case reports and acupuncture procedures twice a month. Throughout the trial, the research team will meet regularly (once every month) to discuss the progress, including recruitment, withdrawal, treatment compliance, and adverse events.

## Discussion

At present, the exact pathogenesis of DCI is still in the exploratory stage. The existing research still lacks imaging markers to accurately explain the cognitive decline of DCI patients, and there are differences in different experimental image processing techniques, such as processing accuracy, brain region segmentation methods, and a lack of ways to analyze the brain imaging data of DCI patients from an individual point of view. The brain imaging data of this study will be completed by the individualized Neuroimaging Research Laboratory of Martinos Neuroimaging Center, Massachusetts General Hospital, Harvard Medical School, USA. This laboratory is the first laboratory in the world to apply rs-fMRI technology to draw the map of functional differences within and between individuals and proposes a new successive recursive method to draw the individual brain network map to improve the reliability of the location results and accurately map the unique functional network organization in each topic [25, 26]. In this way, each patient can be “tailored” to study the cognitive function of his or her brain according to the unique characteristics of his or her brain function, thus helping to make an accurate individualized treatment plan.

In addition, there are no exact and effective drugs or methods for the treatment of DCI in modern medicine, mostly starting from improving symptoms and delaying the disease. On the basis of controlling blood glucose and correcting the disorder of lipid metabolism, drugs to improve the function of brain cells are added for combined treatment, but long-term use easily produces side effects. Related studies have confirmed the important role of acupuncture in the prevention and treatment of diabetes and its complications [13, 27]. However, to date, there have been no properly designed randomized, sham-controlled pilot trials at home or abroad to provide clear evidence to prove the effectiveness of acupuncture in the treatment of DCI and to explain its related mechanism. The intervention method of this study was “Adjust Zang-fu and Arouse Spirit” electroacupuncture therapy, which is based on the previous research results of the research group and the TCM etiology and pathogenesis of DCI. It has the functions of harmonizing Zang-fu organs, freeing *qi* and blood, arousing the brain and regulating spirit. In the early stage, the research group applied this acupuncture method to the clinical treatment of DCI, showing a good clinical effect. At the same time, to eliminate the placebo effect of electroacupuncture, a sham electroacupuncture group was set up, but double-blinding could not be achieved because of the particularity of the electroacupuncture operation.

The main evaluation indicators of this study include brain imaging results and two cognitive assessment scales, and the secondary evaluation indicators include blood glucose and blood lipid detection and four related scales. The time point of evaluation included one evaluation before the treatment period, two evaluations in the treatment period and four evaluations in the follow-up period, for a total of seven evaluations. The brain imaging data of the patients were analyzed at the individual level, and the correlations between the brain imaging, scale results, and test results were analyzed. The purpose of this study was to analyze the changes in brain structure and function in patients with DCI and to explore the therapeutic effect of “Adjust Zang-fu and Arouse Spirit” electroacupuncture on DCI and its correlation with the changes in brain structure and function in patients with DCI.

To summarize, this study is funded by the Project supported by the National Natural Science Foundation of China. The study protocol describes a randomized, sham-controlled pilot trial. The results of this study will provide feasible data and basic information for comprehensively carrying out randomized controlled trial (RCT) experiments of DCI in the future and revealing the mechanism of acupuncture.

## Trial status

This trial is currently ongoing. The study commenced on 10 December 2020 and the anticipated end date of the study is 20 December 2023.

## Data Availability

Not applicable.

## Abbreviations

DCI: Diabetic cognitive impairment
fMRI: Functional magnetic resonance imaging
MRS: Magnetic resonance spectroscopy
MoCA: Montreal Cognitive Assessment Scale
CDR: Clinical Dementia Rating
IADL: Instrumental Activities of Daily Living Scale
HIS: Hachinski Ischemic Scale
SAS: Self-Rating Anxiety Scale
SDS: Self-Rating Depression Scale
DM: Diabetes mellitus
T2DM: Type 2 diabetes mellitus
AD: Alzheimer’s disease
SPIRIT: Standard Protocol Items: Recommendations for Intervention Trials
STRICTA: Standards for Reporting Interventions in Clinical Trials of Acupuncture
HbA1c: Glycosylated hemoglobin A1
TC: Total cholesterol
TGs: Triglycerides
HDL-C: High-density lipoprotein cholesterol
LDL-C: Low-density lipoprotein cholesterol
CRFs: Case report forms
RCT: Randomized controlled trial
